# Virulence determinants of *Pseudomonas syringae* strains isolated from grasses in the context of a small type III effector repertoire

**DOI:** 10.1186/s12866-014-0304-5

**Published:** 2014-12-04

**Authors:** Alexey Dudnik, Robert Dudler

**Affiliations:** Institute of Plant Biology, University of Zurich, Zollikerstrasse 107, Zurich, 8008 Switzerland; Present address: Novo Nordisk Foundation Center for Biosustainability, Technical University of Denmark, Kogle Allé 4, Hørsholm, 2970 Denmark

**Keywords:** *Pseudomonas syringae*, Syringolin A, *Triticum aestivum*, Type III secretion system

## Abstract

**Background:**

*Pseudomonas syringae* is pathogenic to a large number of plant species. For host colonization and disease progression, strains of this bacterium utilize an array of type III-secreted effectors and other virulence factors, including small secreted molecules such as syringolin A, a peptide derivative that inhibits the eukaryotic proteasome. In strains colonizing dicotyledonous plants, the compound was demonstrated to suppress the salicylic-acid-dependent defense pathway. Here, we analyze virulence factors of three strains colonizing wheat (*Triticum aestivum*): *P. syringae* pathovar *syringae* (*Psy*) strains B64 and SM, as well as *P. syringae* BRIP34876. These strains have a relatively small repertoire of only seven to eleven type III secreted effectors (T3Es) and differ in their capacity to produce syringolin A. The aim of this study was to analyze the contribution of various known virulence factors in the context of a small T3E repertoire.

**Results:**

We demonstrate that syringolin A production enhances disease symptom development upon direct infiltration of strains into wheat leaves. However, it is not universally required for colonization, as *Psy* SM, which lacks syringolin biosynthesis genes, reaches cell densities comparable to syringolin A producer *P. syringae* BRIP34876. Next, we show that despite the small set of T3E-encoding genes, the type III secretion system remains the key pathogenicity determinant in these strains, and that phenotypic effects of deleting T3E-coding genes become apparent only when multiple effectors are removed.

**Conclusions:**

Whereas production of syringolin A is not required for successful colonization of wheat leaves by *P. syringae* strains, its production results in increased lesion formation. Despite the small number of known T3Es encoded by the analyzed strains, the type III secretion system is essential for endophytic growth of these strains.

**Electronic supplementary material:**

The online version of this article (doi:10.1186/s12866-014-0304-5) contains supplementary material, which is available to authorized users.

## Background

The genus *Pseudomonas* of the Gram-negative γ-proteobacteria includes a number of species which directly or indirectly influence our everyday life. Members of the genus are metabolically versatile and are associated with various ecological niches and life styles [1]. It includes soil bacteria with biocontrol properties (*P. fluorescens*, *P. putida*, and *P. chlororaphis*) [1-3], opportunistic human pathogens such as *P. aeruginosa* and *P. stutzeri* [4,5], as well as the plant-pathogenic species *P. fuscovaginae*, *P. marginalis*, and *P. syringae* [1,6,7]. Besides *P. aeruginosa*, *P. syringae* is the second best-studied member of this genus, mainly due to its economic impact, but also because of its high genetic and metabolic flexibility that results in a variety of induced disease types and colonized hosts species [8]. The species also serves as a model for plant-pathogen interaction research, mainly with regard to the type III secretion system (T3SS) and effector function [9], plant defense signaling, and gene regulation [10,11].

The T3SS is a complex structure which is used by a number of animal and plant pathogens to deliver so-called effector proteins into cells of their host [12,13]. The type III-translocated effectors (T3Es) are in turn modulating the target cells in a variety of ways. For example, they can suppress defense and other signaling cascades, modify cytoskeleton structure and gene transcription, or interfere with intracellular trafficking [14,15]. The majority of characterized T3Es from *P. syringae* were demonstrated to be involved in the suppression of plant immune responses [16-18]. This is achieved in a variety of ways, and several effectors were demonstrated to degrade components of a defense signaling pathway either directly [19,20] or by ubiquitylation [21]. Other T3Es are known to interfere with signaling either by covalently modifying one of the mitogen-activated protein (MAP) kinases [22], or by inhibiting its kinase activity [23]. Effectors were also shown to inhibit callose deposition [24] and production of reactive oxygen species [25]. It is not uncommon for different effectors to target the same defense pathway, or even the same component within the pathway, thus having a redundant function [14,16,26]. The T3E composition is the key element determining host specificity [27]. Nonetheless, due to functional redundancy, strains isolated from the same host often show variability in their effector sets [28-30].

Apart from T3Es, *P. syringae* strains often produce other substances which promote virulence. One such group of compounds comprises the phytotoxins [31]. Based on their targets and mode of action the phytotoxins can be subdivided into compounds that specifically induce chlorosis or necrosis and general plant defense response suppressors. The macrolactam syringolin A belongs to the second group and was demonstrated to be a proteasome inhibitor [32]. Proteasome-mediated protein degradation is an essential part in a number of hormone-based signaling pathways in plants, including SA-mediated defense signaling [33]. Syringolin A and its minor variants are the products of a mixed non-ribosomal peptide synthetase (NRPS)/polyketide synthetase (PKS) encoded by the *sylA-E* gene cluster of certain *P. syringae* strains [34,35]. It was first discovered in *P. syringae* pathovar *syringae* (*Psy*) B301D-R [34] and has so far been exclusively found among phylogroup II strains of this species [36]. The compound irreversibly inhibits all three catalytic activities of the eukaryotic proteasome [32]. Syringolin A was shown to suppress SA-mediated defense signaling and to counteract stomatal immunity in bean (*Phaseolus vulgaris*) and *Arabidopsis* [37]. Disruption of syringolin A production was demonstrated to result in reduced lesion formation on bean [32] and in diminished wound entry and lesion spreading on *Nicotiana benthamiana* [38]. In addition, syringolin A production has recently been demonstrated in a non-pathogenic strain of *Rhizobium* sp. isolated from eastern cottonwood (*Populus deltoides*), where the compound might potentially play a role in root colonization [39].

In order to fully understand the mechanisms through which syringolin A acts on plant cells and to determine its other potential roles in pathogenesis of *P. syringae*, we were aiming at establishing an infection model for a strain producing this compound in the well-studied model plant *Arabidopsis thaliana*, for which various genetic and “omics”-based tools exist. However, all our attempts to stably transform the *sylA-E* gene cluster into *P. syringae* pv. *tomato* (*Pto*) DC3000, a strain pathogenic to *A. thaliana*, were not successful. Therefore, we decided to explore a different pathosystem involving one of the most important crop plants, common wheat (*Triticum aestivum*), for which we had available several independent isolates naturally containing the syringolin biosynthesis gene cluster (*P. syringae* pv. *syringae* (*Psy*) B64 [40], *P. syringae* BRIP34876, and *P. syringae* BRIP34881 [41]), as well as a strain naturally lacking it (*Psy* SM [42]). It should be noted that both BRIP34876 and BRIP34881 were originally isolated from barley, but are as well able to cause disease on wheat [41].

The genomes of the four above-mentioned strains have been sequenced [40-42], and based on multilocus sequence typing (MLST) analysis, they belong to phylogenetic clade II [43]. Genome analysis has also revealed that all four strains encode a relatively small complement of known T3Es: eleven effectors in BRIP34876 and BRIP34881, ten in *Psy* B64, and seven in *Psy* SM, as compared to 39 effectors in *P. syringae* pv. *tomato* DC3000 [36,43]. A relatively small number of T3Es as well as a reduced HrpL regulon are the two described properties of clade II strains [36,44]. Hence, it is likely that these bacteria rely on non-type III secreted molecules to a greater extent than *P. syringae* strains from other clades. Having strains with a small set of T3Es provides an opportunity for determining roles of individual effectors due to presumably reduced functional overlap, as well as for pinpointing the most important defense pathways that need to be suppressed in order to allow disease progression. Therefore, this work focuses on characterization of virulence determinants in *P. syringae* strains with a small repertoire of type III effectors in wheat.

## Results

### Syringolin production shows strain-specific effects on symptom development in wheat

**Figure 1:**
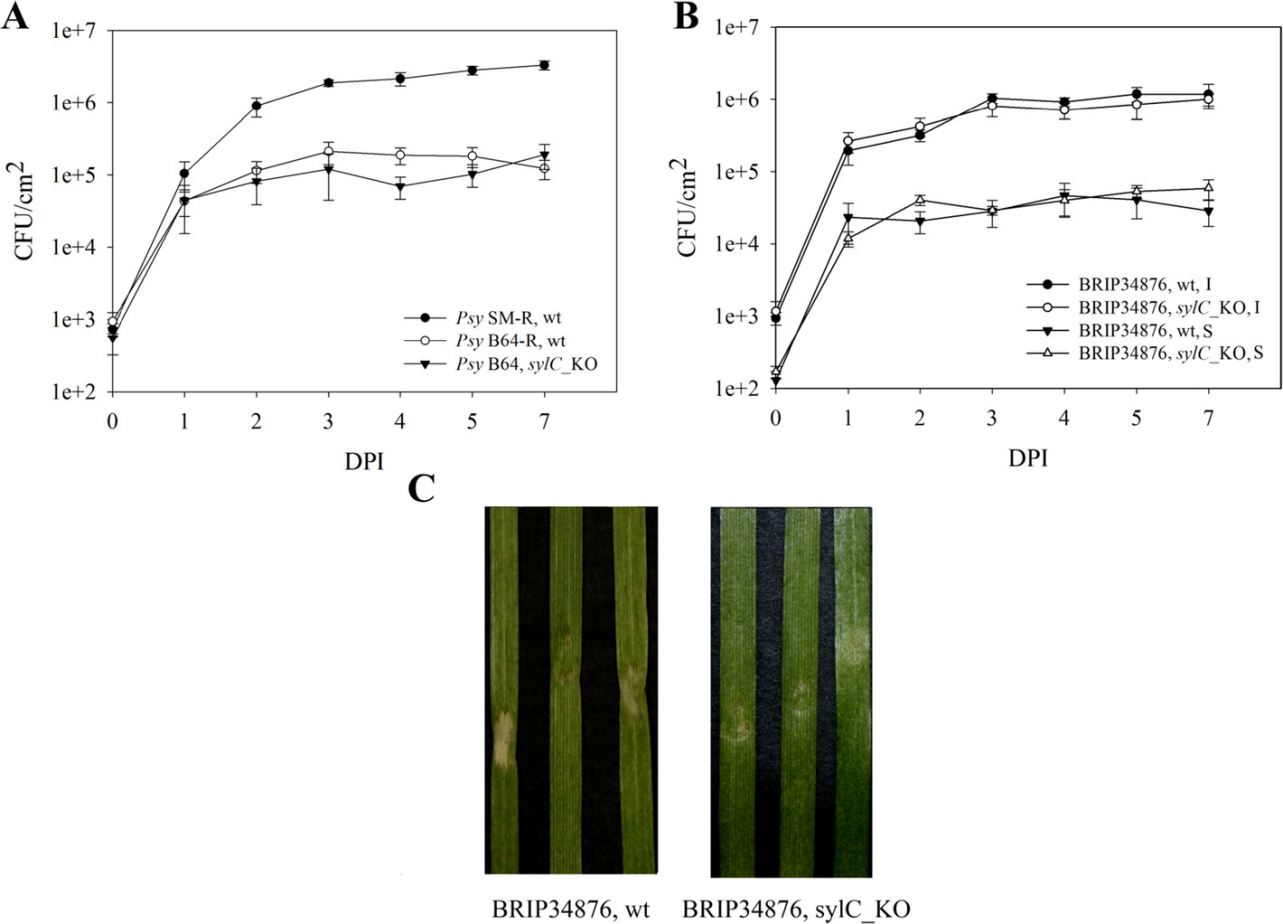
**Endophytic population growth of** ***P. syringae*** **strains in leaves of 10-day-old wheat plants. (A)** Growth of wild-type (wt) *Psy* SM-R, *Psy* B64-R and mutant *Psy* B64-R *sylC*_KO strains after leaf infiltration. **(B)** Growth of wild-type (wt) BRIP34876 and syringolin A-negative mutant BRIP34876 *sylC*_KO after infiltration (I) and surface inoculation (S). CFU, colony forming units; DPI, days post inoculation. Error bars represent standard error of the mean (n = 5 for wild type *Psy* SM-R and *Psy* B64-R, n = 3 for other strains). **(C)** Disease symptoms caused by BRIP34876 wt (left) and BRIP34876 *sylC*_KO (right) photographed 7 DPI.

In order to search for a potential role of syringolin A in the infection process on wheat, wild type (wt) *Psy* SM, *Psy* B64, and BRIP34876 were assayed. The latter two strains contain the *sylA-E* gene cluster (PssB64_04155-PssB64_04151 and A979_11049-A979_11069 respectively), while the first one does not contain it in the genome. *Psy* B64 and BRIP34876 were compared with their respective *sylC* knock-out mutants that are deficient in syringolin A production (*Psy* B64 *sylC*_KO and BRIP34876 *sylC*_KO). Based on genome sequence comparison, strains BRIP34876 and BRIP34881 are nearly identical [43], and therefore, only BRIP34876 was used. In addition, since surface inoculation is a more natural way of infection, as well as because syringolin A was demonstrated to be involved in counteracting stomatal immunity in dicot species [37], the strains were analyzed using both infiltration and surface inoculation. Note that all experiments were carried out using spontaneous rifampin-resistant mutants of *Psy* SM and *Psy* B64 (indicated with an “R”) that are otherwise identical to the original isolates. The results are presented in Figure 1.

When infiltrated directly into the leaf mesophyll, *Psy* B64-R and its *sylC*-negative derivative did not show significant growth differences (Figure 1A and Additional file 1: Table S4). Infiltrated leaves remained green and completely asymptomatic with both strains (Additional file 1: Figure S1A and S1B). Similarly, wild type BRIP34876 and the corresponding *sylC_KO* mutant also showed equal endophytic growth under these conditions (Figure 1B and Additional file 1: Table S4). However, in contrast to *Psy* B64-R, BRIP34876-infiltrated leaves exhibited more tissue damage and slightly higher level of chlorosis, which was less pronounced in the *sylC*-deficient mutant (Figure 1C). Interestingly, when infiltrated into leaf mesophyll, *Psy* SM-R showed a growth profile comparable to that of BRIP34876 despite the lack of the *sylA*-*E* gene cluster. However, this strain caused a different set of symptoms, namely small lesions spread throughout the entire infiltrated area, without a pronounced chlorosis (Additional file 1: Figure S1C). When bacteria were inoculated by dipping, no difference in both endophytic growth and visible symptom formation was observed between the wild type and the *sylC* knock-out mutants of both BRIP34876 (Figure 1C and data not shown) and *Psy* B64-R (data not shown). Moreover, after surface inoculation, none of the strains was able to grow to the same cell densities that were observed upon direct infiltration.

### The T3SS is the key pathogenicity determinant

**Figure 2:**
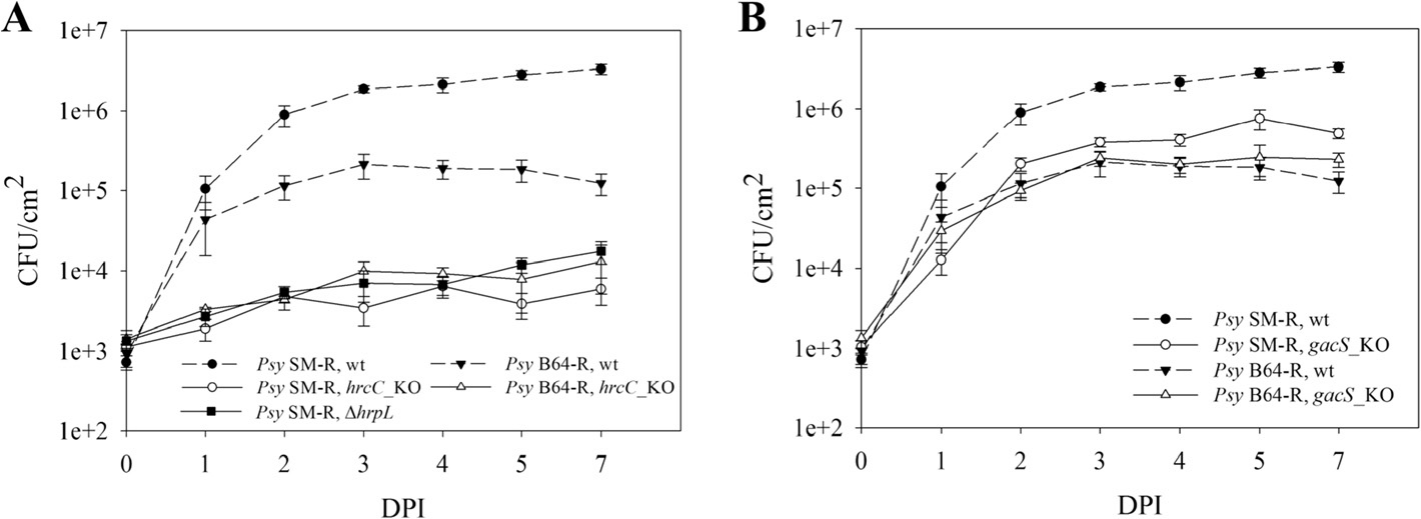
**Influence of the T3SS and the GacA-GacS global regulatory system on endophytic growth. (A)** Endophytic growth after leaf infiltration of T3SS-negative *hrcC* mutants (*hrcC_*KO) in *Psy* SM-R and *Psy* B64-R as well as the T3SS-regulatory deletion mutant *ΔhrpL* in *Psy* SM-R was compared with the respective wild-type (wt) strains. **(B)** Endophytic growth comparison after leaf infiltration of *gacS* knockout mutants (*gacS_*KO) and wild-type strains (wt) in *Psy* SM-R and *Psy* B64-R strains. The data for the wt strains are the same as in **(A)**. DPI, days post infection; CFU, colony forming units. Error bars indicate the standard error of the mean (n = 4 for *gacS_KO* strains, n = 3 for other strains).

A relatively small number of encoded T3Es is a property described for phylogroup II strains [36]. However, the wheat and barley isolates appear to have a particularly minimized repertoire of the T3Es: eleven in BRIP34876 and BRIP34881, ten in *Psy* B64-R, and seven in *Psy* SM-R [43]. Therefore, it is possible that these strains rely to a larger extent on non-type III secreted molecules, such as e.g. phytotoxins. In order to test this hypothesis, T3SS-deficient mutants were generated by knocking out the *hrcC* gene, which encodes an essential structural component of the T3SS [45]. *Psy* B64-R and *Psy* SM-R were chosen for this analysis because the two strains had the smallest number of T3Es among the strains available to us. Interestingly, knocking out the T3SS in both strains nearly abolished their ability to reproduce *in planta*, thus rendering them non-pathogenic (Figure 2A and Additional file 1: Table S4). The same result was observed upon deletion of the *hrpL* gene in *Psy* SM-R (Figure 2A and Additional file 1: Table S4), which encodes a transcriptional regulator controlling the expression of the T3SS, as well as of several other non-T3SS-associated genes [44,46]. This shows that the type III secretion system still remains essential for the infection progress and that the small effector repertoire is sufficient to allow mesophyll colonization.

Regulation of the T3SS in *P. syringae* differs between strains. In the model strain *P. syringae* pv. *tomato* (*Pto*) DC3000, it is under control of the GacA/GacS two-component system [47]. This two-component system is a global regulator in *P. syringae* and other pseudomonads [48], controlling, among others, quorum sensing, motility, phytotoxin and protease production [47,49,50]. In contrast, in *P. syringae* pv. *syringae* B728a, T3SS regulation appears to be uncoupled from the regulation by GacS [47,51,52]. Both *Psy* SM-R and *Psy* B64-R are more closely related to *Psy* B728a than to *Pto* DC3000 [43], and therefore it is likely that the wheat isolates also have the T3SS functioning independently of GacA/GacS regulation. Such a setting would present a good opportunity to study an influence of non-type III secreted virulence factors, and therefore knock-out mutants of the *gacS* gene were constructed in the two strains.

When tested for endophytic growth, *Psy* SM-R *gacS*_KO showed an approximately eight-fold reduction in endophytic growth after infiltration (Figure 2B and Additional file 1: Table S4). In addition, *Psy* SM-R *gacS*_KO infiltrations resulted in a minor reduction of lesion numbers (Additional file 1: Figure S1D). Surprisingly, knocking out the *gacS* gene in *Psy* B64-R had no effect at all on mesophyll colonization of this strain (Figure 2B). Spontaneous inactivation of the GacA/GacS two-component system is a well-known phenomenon among the pseudomonads [53,54], and therefore could be a potential reason for the observed phenotype. To further investigate this, *Psy* B64-R was tested for syringolin A production using our rice assay (*pir7b* expression induction in rice in the presence of syringolins [35]), as well as for protease production by growing the strain on milk-agar. Both tests yielded negative results (Additional file 1: Figure S2), thus supporting the hypothesis that the GacA/GacS system in *Psy* B64-R is not active, in spite of the fact that, based on our genome sequence analysis, the respective genes appear to be intact. The lack of a functional GacA/GacS two-component system would explain the observed differences in overall endophytic growth of the wild type *Psy* SM-R and *Psy* B64-R (Figure 1A). In addition, this would also explain the lack of any visible lesions on *Psy* B64-infiltrated leaves, as *gacA*- or *gacS*-negative mutants in *P. syringae* are known to show a significant reduction in disease symptom intensity [47,51].

### Importance of individual T3Es

Because of the small effector repertoire and ease of genetic manipulation we decided to further characterize *Psy* SM-R. The genome of this strain encodes the following seven known T3Es: AvrE1, HopM1, HopI1, HopAA1, HopBA1, HopAZ1, and HopA2 [42]. Only two of these are well-characterized: HopM1 targets ADP ribosylation factor-guanine nucleotide exchange factor (ARF-GEF) proteins for degradation [55,56], and HopI1 is involved in the degradation of the Hsp70 chaperone [57]. Out of the remaining ones, AvrE1 is hypothesized to mimic activated G-proteins [58], while HopAA1 is a necrosis-inducing effector with a putative GTPase-activating protein (GAP) domain [59] that was demonstrated to enhance epiphytic growth and survival in tomato [60]. However, no exact molecular mechanism has been described so far for the latter two. These four effectors are found in almost all of the currently sequenced *P. syringae* strains [36,61], and although disrupted or truncated in some strains, they are likely involved in the modulation of conserved host defense pathways. Moreover, mutants of these four “core” effectors have been characterized in terms of the impact on growth and disease progression [62,63]. Therefore, we decided to focus on the three remaining effectors: HopA2, HopAZ1, and HopBA1. In order to investigate the importance of the selected effectors, individual markerless gene deletion mutants were constructed. Furthermore, all possible combinations of double mutants and the triple mutant were generated as well.

**Figure 3:**
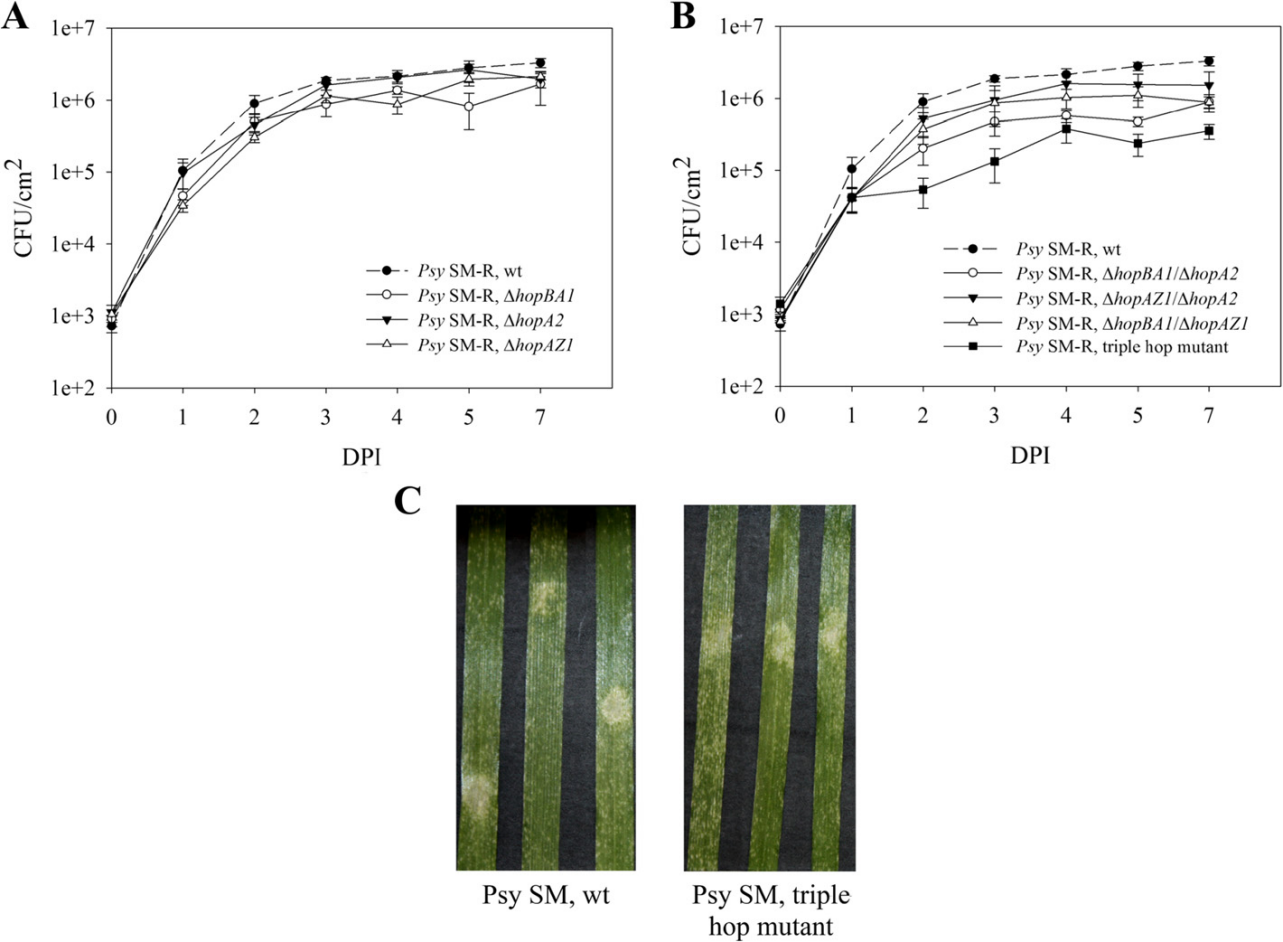
**Role of the non-core T3Es in colonization of wheat leaves by** ***Psy*** **SM-R.** Endophytic growth after leaf infiltration of **(A)** individual T3E mutants and **(B)** double and the triple T3E deletion mutants as compared to the wild type (wt) *Psy* SM-R. **(C)** Disease symptoms of *Psy* SM-R wt (left) and the triple hop mutant (right). The data for the wt strain is the same as in **(A)**. DPI, days post infection; CFU, colony forming units. Error bars indicate standard error of the mean (n = 3 for all strains).

When infiltrated into wheat leaves, the individual T3E mutants resulted in an insignificant endophytic growth reduction when compared to the wild type (Figure 3A and Additional file 1: Table S4). The double mutants showed a further reduction of endophytic growth, which was most pronounced in the Δ*hopBA1*/Δ*hopA2* strain, where bacterial counts were four to five times smaller as compared to the wild type (Figure 3B and Additional file 1: Table S4). The triple mutant showed an *in planta* growth reduction of on average about 10-fold (Figure 3B and Additional file 1: Table S4). What is also notable is that leaves infiltrated with the triple mutant showed a degree of lesion formation similar to the wild type *Psy* SM-R, despite the reduced bacterial density (Figure 3C).

## Discussion

While it is generally assumed that phytotoxin production in *P. syringae* is not absolutely required for the infection process, these compounds have been demonstrated to enhance disease progression and symptom development [31,64]. Here, we have demonstrated that, when produced in wild type amounts, syringolin promotes lesion formation on wheat. This also confirms the previous findings by Groll and colleagues [32], who reported that lack of syringolin production resulted in reduced lesion formation on bean. Furthermore, we demonstrate that decreased lesion formation is not due to a reduction of the endophytic bacterial density. At present we can only speculate why this is the case. One possible explanation is that presence of syringolin allows a more efficient effector translocation and/or production of necrosis-inducing toxins such as syringomycin, which in turn would result in a higher level of damage to host tissue. What is also interesting, and rather contradictory, is the presence of T3Es that require the proteasome for their function, such as HopM1 [65], in strains that produce syringolin A. It is however possible that production and activities of the two virulence factors are separated in a temporal and/or in a spatial manner during the infection.

Another interesting observation was the effect of the *hopBA1* deletion, which alone, or in combination with the other non-core T3Es, would result in a somewhat lower virulence when compared to the wild type strain and some other mutant combinations (Figure 3 and Additional file 1: Table S4). Moreover, *hopBA1* is common to all currently published phylogroup II *P. syringae* strains isolated from grasses (family Poaceae) [43]. Thus, based on the obtained results, this effector indeed appears to play an important role in colonization of the wheat leaf mesophyll. Hence, further studies on this protein could provide some interesting insights into its exact role in this process, as well as on Poaceae-specific immunity mechanisms.

It is also apparent from our results that the seven identified T3Es are indeed sufficient for *Psy* SM-R to allow proper endophytic growth on wheat. Based on the growth of the *gacS_*KO mutant, it appears that the relative contribution of the GacA/GacS regulon to the overall virulence is about 30%, with a further approximately 30% being contributed by the three non-core T3Es, and the rest being potentially attributed to the four core T3Es. This, however, does not completely exclude the possibility that the strain possesses other, so far unknown, effectors, but if indeed present, their contribution to the overall virulence appears negligible. This finding is in good agreement with previously published work on the minimal effector repertoire required by *Pto* DC3000 to grow on *N. benthamiana* [26], where the authors determined that only eight T3Es out of about three dozen were sufficient to reach bacterial cell counts comparable to those of the wild type strain. This work also demonstrated that in some cases, addition of a single effector resulted only in a minor increase of endophytic growth. Here, a similar trend was observed, as the analyzed single T3E mutants only showed a minor decrease in endophytic bacterial growth. This suggests that despite the small effector number, there is still some degree of redundancy between those. It should be noted that currently it is not possible to completely exclude that the reported phenotypes are the product of spontaneous secondary mutations, but technical challenges prevented us from performing complementation studies.

Nonetheless, even though the seven effectors allow *Psy* SM-R to reach a relatively high population density, it seems that their activity could be insufficient to fully suppress host immunity. The damage to leaf tissue, which was at first assumed to be disease symptoms, appeared in somewhat higher amounts on leaves infiltrated with the triple hop mutant, even though lower population densities were reached by this strain (see Figure 3C). One possible explanation for this observation could be that upon reaching a certain cell density, even the wild type strain was not fully able to down-regulate the host immune response. The triple hop mutant is further impaired in this ability, and thus, the lesions appear at even lower bacterial densities. However more evidence is needed to draw a definitive conclusion.

## Conclusions

This work provides evidence that syringolin A production contributes to virulence and visual symptom development of some *P. syringae* strains on wheat. Interestingly however, production of this compound does not result in increased endophytic bacterial densities as compared to syringolin-deficient strains. What was also unexpected was that there was no difference in entry efficacy between the wild type BRIP34876 and its *sylC_*KO mutant when dip-inoculation was used.

Despite the small number of T3Es found in the genomes of the analyzed strains, knocking out the T3SS resulted rendered these strains completely avirulent. The contribution of individual non-core effectors appears to be minor, at least in the case of *Psy* SM-R. Interestingly however, the degree by which individual mutations (Δ*hopBA1*, Δ*hopA2*, or Δ*hopAZ1*) affected the endophytic populations was different, and the largest decrease was observed for the *hopBA1*-deficient strain.

## Methods

### Bacterial strains and growth conditions

Bacterial strains and plasmids used in this study are listed in Additional file 1: Table S1. Unless otherwise stated, bacteria were grown in Lysogeny Broth (LB) medium (tryptone 10 g l^−1^, yeast extract 5 g l^−1^, and NaCl 5 g l^−1^) containing the appropriate amount of antibiotics: ampicillin (50 μg ml^−1^), chloramphenicol (25 μg ml^−1^), gentamicin (50 μg ml^−1^), rifampin (50 μg ml^−1^), or tetracycline (15 μg ml^−1^) at 28°C (*P. syringae*) or 37°C (*E. coli*) under constant agitation (220 rpm). For solid media 1.5% agar was used.

### Generation of pJQ200KSΔP*lac* and generation of plasmid insertion mutants

If not stated otherwise, standard procedures were used [66,67]. To avoid a possible polar effect in plasmid insertion mutants due to the *lac* promoter of the suicide vector pJQ200KS which could be observed after integration of mutagenic constructs into the genome, the corresponding DNA fragment was removed by digesting the vector with *SphI* and *ApaI*. The vector fragment was blunted using T4 DNA polymerase in the presence of excess dNTPs and re-ligated overnight at 4°C. The *ApaI* site is reconstituted in the resulting plasmid.

To generate an insertional gene knock-out mutant, an approximately 700-bp-long fragment of a target gene was amplified using the respective P1 and P2 primers (Additional file 1: Table S2). The primers were designed such that the inserted construct would disrupt a predicted conserved domain, where available. The fragment was then cloned into one of the suicide vectors, pJQ200KSΔP*lac* or pME3087, using respective restriction enzymes, and transformed into *E. coli* XL-1 Blue. After verification by sequencing, the construct was mobilized into *P. syringae* by tri-parental mating using *E. coli* HB101 (pRK600) as a helper strain. In order to generate the *sylC*_KO mutant of BRIP34876, the respective construct was first transformed into *E. coli* ST18, which then served as a donor in bi-parental mating. The *sylC*_KO mutant of *Psy* B64 was generated using bi-parental mating with *E. coli* S17-1 (pME3087-PS3) [34]. The location of the integrated construct was verified by PCR using the respective ch1 primer and either pJQ200KS_B1_R when pJQ200KSΔP*lac* was used, or pr_3087 H3 when pME3087 was used (See Additional file 1: Table S2 for a complete list of primers and Additional file 1: Table S3 for a list of target genes).

### Generation of markerless in-frame gene deletion mutants

Two approximately 700 bp-long fragments originating from upstream and downstream regions, respectively, of target gene’s open reading frame (ORF) were amplified using the respective primers (Additional file 1: Table S2; primers P1 and P2 amplify the corresponding upstream fragments, primers P3 and P4 amplify the corresponding downstream fragments). The upstream and downstream fragments were joined by overlap extension PCR using primers P1 and P4, cloned into the suicide vectors pJQ200KSΔP*lac* using respective restriction enzymes, and transformed into *E. coli* XL-1 Blue. After verification by sequencing, the construct was mobilized into *P. syringae* by tri-parental mating using *E. coli* HB101 (pRK600) as a helper strain. Single recombinants of *P. syringae*, which were selected on plates containing the suitable antibiotic, were then grown on LB plates supplemented with 10% sucrose in order to select for double recombinants. Deletion of the target sequence was verified by PCR using the respective oriA and oriB primers, followed by sequence determination. See Additional file 1: Table S2 for a complete list of primers and Additional file 1: Table S3 for a list of target genes. The resulting gene deletions retained an open reading frame (ORF) of eighteen to thirty bases, including the start and the stop codons.

### Plant growth and infection assays

Common wheat (*Triticum aestivum*) cultivar Chinese Spring was grown under 16 hours light/8 hours dark conditions at 20°C during the light phase and 18°C during the dark phase without controlled humidity. For infiltration of 10-11-day-old plants, bacteria were grown overnight in 5 ml of LB medium. The culture was diluted the next day 1:25 in a total volume of 5 ml, grown further until an OD_600_ of 0.4-0.8 was reached, centrifuged at 2500 × g, and re-suspended in sterile dH_2_O. The suspension was diluted to 10^6^ colony-forming units (CFU) per ml (OD_600_ 0.002) and infiltrated into primary leaves using a 3 ml needleless syringe. For surface (dip) inoculation, the overnight culture was diluted 1:25 in a total volume of 100 ml. The culture was further grown until an OD_600_ of 0.4-0.8 was reached, at which point it was harvested by centrifugation at 2500x g. The cells were washed once, and then re-suspended in dH_2_O. The suspension was then diluted to 10^8^ CFU/ml (OD_600_ 0.2), and 0.03% of the surfactant Silwet L-77 (Leu + Gygax AG, Birmenstorf, Switzerland) was added. Inoculation was performed by dipping primary leaves into the suspension for approximately 30 seconds.

Independently of the inoculation method used, counting of endophytic bacteria was performed as follows: a 3 cm-long leaf segment was cut out of the infiltrated area, surface-sterilized in EtOH for 15 seconds, and washed in dH_2_O. Three leaf fragments infiltrated with the same strain were pooled together and macerated in 200 μl of sterile dH_2_O using a sterilized pestle. Once only the major veins remained visible, 800 μl of dH_2_O were added, and a 10-fold dilution series was generated. 50 μl of each dilution were plated onto LB-agar and incubated either for 42–44 hours at room temperature (*Psy* SM-R and *Psy* B64-R), or for 24–26 hours at 28°C (BRIP34876). Each tested strain was assayed at least three times independently.

### Protease and syringolin A production assays

Protease production was measured by growing bacteria on 5% skim milk-agar plates for five days at 20°C. Syringolin A production was assayed by monitoring *pir7b* transcript accumulation in infiltrated rice leaves as described by Ramel and colleagues [35].

### Ethics statement

Experimental research on plants has been carried out with accordance to institutional and Swiss national regulations. No collection of plant material, field work, or any type of work involving transgenic plants has been carried out within the frame of this project. No research involving human or animal subjects, human material, or human data has been done as a part this project. No clinical trials or any type of medically-related work was performed. Thus, no additional permissions were required from ethics or any other committees.

## Additional file

Additional file 1: Figure S1. Wheat leaves photographed 7 days after infiltration with **(A)** Psy B64-R wt, **(B)** Psy B64 sylC_KO, **(C)** Psy SM-R wt, **(D)** Psy SM-R gacS_KO. **Figure S2. (A)** RNA gel blot depicting pir7b gene transcript levels in RNA extracted from rice leaves 16h after infiltration with P. syringae strains. **(B)** Test for protease production using milk-agar plates. **Table S1.** List of strains and plasmids used in this study. **Table S2.** The list of primers used in this study. **Table S3.** List of the mutated and other genes mentioned in this work with the corresponding locus tags. **Table S4.** Statistical evaluation of the endpoint growth kinetics data on the evaluated strains.

## Abbreviations

*Psy*: *Pseudomonas syringae* pathovar *syringae*
Pv: Pathovar
T3SS: Type III secretion system
T3E: Type III effector
SA: Salicylic acid
NRPS/PKS: Non-ribosomal peptide synthetase/polyketide synthetase
DPI: Days post inoculation
CFU: Colony-forming units
