# Supplementary material for: Virulence determinants of Pseudomonas syringae strains isolated from grasses in the context of a small type III effector repertoire
Source: BMC Microbiol. 2014 Dec 4;14:304. doi: 10.1186/s12866-014-0304-5 (PMC4262972; doi:10.1186/s12866-014-0304-5)
Supplement: Additional file 1: Figure S1. — Wheat leaves photographed 7 days after infiltration with (A) Psy B64-R wt, (B) Psy B64 sylC_KO, (C) Psy SM-R wt, (D) Psy SM-R gacS_KO. Figure S2. (A) RNA gel blot depicting pir7b gene transcript levels in RNA extracted from rice leaves 16h after infiltration with P. syringae strains. (B) Test for protease production using milk-agar plates. Table S1. List of strains and plasmids used in this study. Table S2. The list of primers used in this study. Table S3. List of the mutated and other genes mentioned in this work with the corresponding locus tags. Table S4. Statistical evaluation of the endpoint growth kinetics data on the evaluated strains. [file 12866_2014_304_MOESM1_ESM.pdf]

Virulence determinants of *Pseudomonas syringae* strains isolated from grasses in the context of a small type III effector repertoire

Alexey Dudnik[1§*] **and Robert Dudler**[1]

[1] Institute of Plant Biology, University of Zurich, Zollikerstrasse 107, 8008, Zurich, Switzerland

[§] Corresponding author

* Present address: Novo Nordisk Foundation Center for Biosustainability, Technical University of Denmark, Kogle Allé 4, 2970, Hørsholm, Denmark

E-mail addresses:

AD: adud@biosustain.dtu.dk

RD: rdudler@botinst.uzh.ch

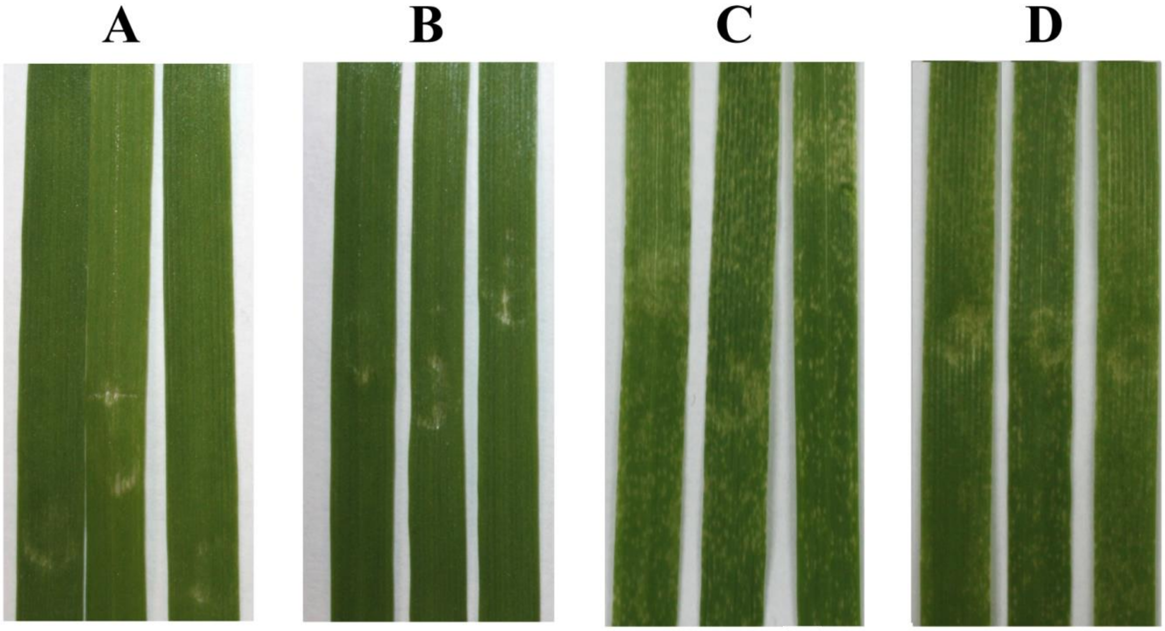

Figure S1. Wheat leaves photographed 7 days after infiltration with (**A**) *Psy* B64-R wt, (**B**) *Psy* B64 *sylC*_KO, (**C**) *Psy* SM-R wt, (**D**) *Psy* SM-R *gacS*_KO.

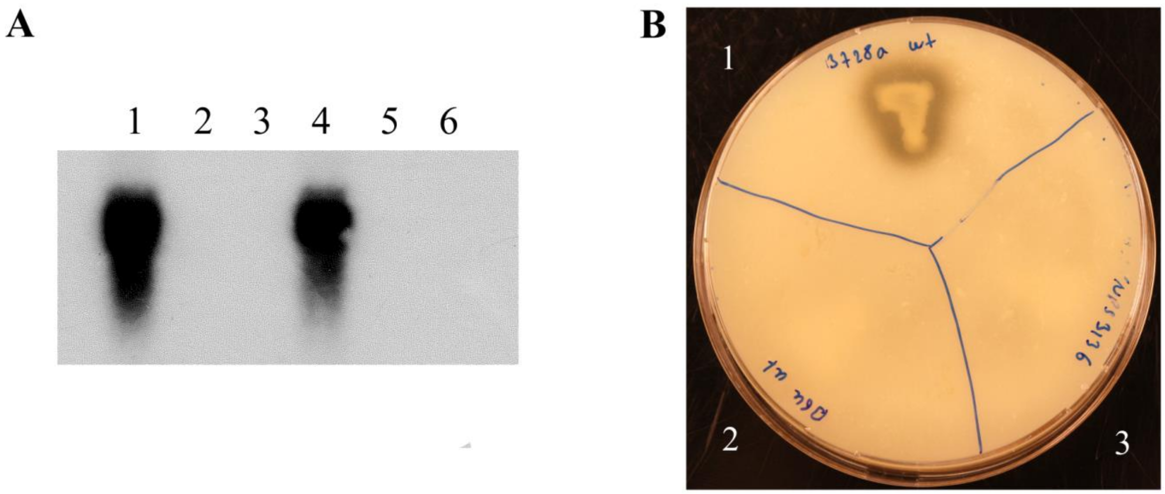

Figure S2. (**A**) RNA gel blot depicting *pir7b* gene transcript levels in RNA extracted from rice leaves 16h after infiltration with *P. syringae* strains. A radiolabeled *pir7b* hybridization probe was used. *Pir7b* transcripts accumulate exclusively in the presence of syringolin A (Ramel *et al.*, 2009). (1) and (4) *Psy* B301D-R wt, (2) and (5) *Psy* B64-R wt, (3) and (6) *Psy* B64-R *gacS*_KO. (**B**) Test for protease production using milk-agar plates. The halo occurs due to protease secretion. (1) Psy B728a wt, (2) Psy B64-R wt, (3) NPS3136 (Psy B728a gacS::Tn5).

Table S1. List of strains and plasmids used in this study

Strain or Plasmid	Genotype, relevant characteristics	Source or Reference
Pseudomonas syringae		
Psy SM	Pathovar *syringae*, wild type strain, isolated from wheat, Chl^R	[1]
Psy SM-R	Spontaneous Rif^R mutant of *Psy* SM	This study
Psy SM-R, *gacS*_KO	*gacS* knock-out mutant of *Psy* SM-R, Gent^R	This study
Psy SM-R, *hrcC*_KO	*hrcC* knock-out mutant of *Psy* SM-R	This study
Psy SM-R, Δ*hrpL*	*hrpL* deletion mutant of *Psy* SM-R	This study
Psy SM-R, Δ*hopBA1*	*hopBA1*deletion mutant of *Psy* SM-R	This study
Psy SM-R, Δ*hopA2*	*hopA2* deletion mutant of *Psy* SM-R	This study
Psy SM-R, Δ*hopAZ1*	*hopAZ1*deletion mutant of *Psy* SM-R	This study
Psy SM-R, Δ*hopBA1*/Δ*hopA2*	*hopBA1*/*hopA2* deletion mutant of *Psy* SM-R	This study
Psy SM-R, Δ*hopBA1*/Δ*hopAZ1*	*hopBA1*/*hopAZ1* deletion mutant of *Psy* SM-R	This study
Psy SM-R, Δ*hopAZ1*/Δ*hopA2*	*hopAZ1*/*hopA2* double deletion mutant of *Psy* SM-R	This study
Psy SM-R, triple hop mutant	*hopBA1*/*hopA2*/*hopAZ1* triple deletion mutant of *Psy* SM-R	This study
Psy B64	Pathovar *syringae*, wild type strain, isolated from wheat	[2]
Psy B64, *sylC*_KO	*sylC* knock-out mutant of *Psy* B64, Tet^R	This study
Psy B64-R	Spontaneous Rif^R mutant of *Psy* B64	This study
Psy B64-R, *gacS*_KO	*gacS* knock-out mutant of *Psy* B64-R, Gent^R	This study
Psy B64-R, *hrcC*_KO	*hrcC* knock-out mutant of *Psy* B64-R, Gent^R	This study
BRIP34876	Wild type, isolated from barley	[3]
BRIP34876, *sylC*_KO	*sylC* knock-out mutant of BRIP34876, Gent^R	This study
Psy B301D-R	Pathovar *syringae*, wild type strain, isolated from pear, Rif^R	[4]
Psy B728a	Pathovar *syringae*, wild type strain, isolated from bean, Rif^R	[5]
NPS3136	*gacS* knock-out mutant of *Psy* B728a, Rif^R/Kan^R	[6]
Escherichia coli		
XL-1 Blue	*endA*1 *gyrA*96(nal^R) *thi*-1 *recA*1 *relA*1 *lac* *glnV*44 F'[::Tn10 *proAB*$^+$ *lacI*^{q} Δ(*lacZ*)M15] *hsdR*17(r_K^- m_K^+)	[7]
HB101 (pRK600)	F$^-$ *mcrB* *mrr* *hsdS*20(r_B^- m_B^-) *recA*13 *leuB*6 *ara*-14 *proA*2 *lacY*1 *galK*2 *xyl*-5 *mtl*-1 *rpsL*20(Sm^R) *glnV*44 λ$^-$ /Cm^R Nm^S, pRK2013 Nm^R::Tn9	[8]
SURE	*endA*1 *glnV*44 *thi*-1 *gyrA*96 *relA*1 *lac* *recB* *recJ* *sbcC* *umuC*::Tn5 *uvrC* e14- Δ(*mcrCB-hsdSMR-mrr*)171F'[*proAB*$^+$ *lacI*^{q} *lacZ*ΔM15 Tn10]	Stratagene
S17-1	*thi pro hsdR recA* chromosomal RP4 [Tra+ Tc^S Km^S Ap^S]	[9]
ST18	*thi pro hsdR recA hemA* chromosomal RP4 [Tra+ Tc^S Km^S Ap^S]	[10]
Plasmids		
pJQ200KS	Suicide vector, p15A ori, Gent^R	[11]
pJQ200KSΔP*lac*	pJQ200KS, Δ*lac* promoter	This study
pME3087	Suicide vector, ColE1 ori, Tet^R	[12]

Table S2. The list of primers used in this study.

Primer	Sequence
BRIP_sylC_KO_P1	TAT GGA TCC GCT CAG GAA ACA GCG ACT TC
BRIP_sylC_KO_P2	ATC AGT CGA CCC GTA CCC TTG ACC AGA CTC
BRIP_sylC_KO_ori	CAG GCT GAA CCA CCA ACT G
gacS_new_P1	TAT GGA TCC AGA ACT GCA ACT GAG CAT CG
gacS_new_P2	ATC AGT CGA CCT GAA GGC CTG GAA CAG TG
gacS_new_ch1	CTC GAT CAG GCG TTT GGA G
hrcC_ins_P1	TAT GGA TCC AGA GCC CGG AAG AGT TTC TC
hrcC_ins_P2	ATC AGT CGA CTG GAC AGT TCT GCC AGC TC
hrcC_ori_ch1	GAG GCT GAG CCG TTA CCT TC
hrpL_new_P1	TAT GGA TCC GAA ACC GCT ACG CCA TAC AG
hrpL_new_P2	CAC TCA GGC GAA GAT CAC AAG ATT CGG GAG CAT
hrpL_new_P3	GAA TCT TGT GAT CTT CGC CTG AGT GAA CAT CTG
hrpL_new_P4	ATC AGT CGA CTC AAG GGA TTG AGG ATC AGC
hrpL_new_ori_A	GTA ATC GCC GAA AGA TCG TC
hrpL_new_ori_B	TTT AAT CAG CGC TTT CAG ACC
hopBA_del_P1	TAT GGA TCC CTC GGC TTT CAT GTC TTG C
hopBA_del_P2	TTA TCC TCG CCA TTT AGC ATA TTA TGT CCT TCG TTG
hopBA_del_P3	ATA TGC TAA ATG GCG AGG ATA ACT AAA GAA AAC C
hopBA_del_P4	ATC AGT CGA CTT GCC AAG GGT GAT GTA GTG
hopBA_oriA	TTA TAA CCC GCG AGA GTT GC
hopBA_oriB	GGT TTG CTG GAC TGG CCT AC
hopA_del_P1	TAT GGA TCC CTG CTA TCC CGG TCA TCG
hopA_del_P2	TAC TCA TTC CAA TAT CGG GTT CAT GAC TCA TCC TC
hopA_del_P3	TGA ACC CGA TAT TGG AAT GAG TAT GAA CTG CTG
hopA_del_P4	ATC AGT CGA CCC AGA ATC TCC AGC TTA CCG
hopA_oriA	AAT CAA GGG CGG CAT AGA C
hopA_oriB	CAT CGA TCA GAA TCG ACG AC
hopAZ-S_del_P1	TAT GGA TCC GTT GTG ACG CAC GAA GTA GC
hopAZ-S_del_P2	CAT CGC GCC ATA AAG GTC ATG GGT ATT TTC CTC
hopAZ-S_del_P3	CCA TGA CCT TTA TGG CGC GAT GGA AGT AAG
hopAZ-S_del_P4	ATC AGT CGA CCT GTA GCT GCC GTA GCA GTC
hopAZ-S_oriA	ACG AAC GCG GTG ATA TTG AC
hopAZ-S_oriB	TCC TCT GTG CTG ACT TGT CG
pJQ200KS_B1_R	TCT AGA ACT AGT GGA TCC
pr_3087 H3	GAG AAA TCA CCA TGA GTG

Table S3. List of the mutated and other genes mentioned in this work with the corresponding locus tags.

Strain	Gene	Locus tag	Mutant type
Psy SM-R	*hrcC*	PssSM_1194	Knock-out*
	hrpL	PssSM_1210	Deletion#
	gacS	PssSM_3753	Knock-out
	hopBA1	PssSM_2782	Deletion
	hopAZ1	PssSM_1866	Deletion
	hopA2	PssSM_1213	Deletion
	avrE‡	PssSM_1182	-
	hopM1‡	PssSM_1180	-
	hopI1‡	PssSM_4439	-
	hopAA1‡	PssSM_1177	-
Psy B64-R	*sylC*	PssB64_04153	Knock-out
	hrcC	PssB64_04549	Knock-out
	gacS	PssB64_02187	Knock-out
BRIP34876	*sylC*	A979_11059	Knock-out

* Inactivation by plasmid insertion

In-frame deletion of the sequence

‡ No mutants of these genes were generated

Table S4. Statistical evaluation of the endpoint growth kinetics data on the evaluated strains.

Comparison	Student's t-test *p-value*	
	At 5 DPI	At 7 DPI
Psy B64-R, wt versus *sylC*_KO	0.2762	0.4584
BRIP34876, wt vs *sylC*_KO, infiltration	0.4688	0.7312
BRIP34876, wt vs *sylC*_KO, dip-inoculation	0.5993	0.2264
Psy SM-R, wt vs *hrcC*_KO	0.0017**	0.0023**
Psy SM-R, wt vs *hrpL*_KO	0.0017**	0.0024**
Psy B64-R, wt vs *hrcC*_KO	0.0380*	0.0455*
Psy SM-R, wt vs *gacS*_KO	0.0030**	0.0038**
Psy B64-R, wt vs *gacS*_KO	0.6302	0.1194
Psy SM-R, wt vs Δ*hopA2*	0.8670	0.1014
Psy SM-R, wt vs Δ*hopBA1*	0.0181*	0.1753
Psy SM-R, wt vs Δ*hopAZ1*	0.1772	0.0709
Psy SM-R, Δ*hopA2* vs Δ*hopBA1*	0.1348	0.7801
Psy SM-R, Δ*hopA2* vs Δ*hopAZ1*	0.5213	0.7723
Psy SM-R, Δ*hopBA1* vs Δ*hopAZ1*	0.1191	0.6263
Psy SM-R, wt vs Δ*hopA2*/Δ*hopBA1*	0.0029**	0.0056**
Psy SM-R, wt vs Δ*hopA2*/Δ*hopAZ1*	0.1717	0.1443
Psy SM-R, wt vs Δ*hopBA1*/Δ*hopAZ1*	0.0175*	0.0048**
Psy SM-R, wt vs triple hop mutant	0.0019**	0.0031**
Psy SM-R, Δ*hopBA1*/Δ*hopA2* vs Δ*hopAZ1*/Δ*hopA2*	0.1625	0.4820
Psy SM-R, Δ*hopBA1*/Δ*hopA2* vs Δ*hopBA1*/Δ*hopAZ1*	0.1527	0.9670
Psy SM-R, Δ*hopBA1*/Δ*hopA2* vs triple hop mutant	0.0902	0.0373*
Psy SM-R, Δ*hopAZ1*/Δ*hopA2* vs Δ*hopBA1*/*ΔhopAZ1*	0.5625	0.4839
Psy SM-R, Δ*hopAZ1*/Δ*hopA2* vs triple hop mutant	0.1058	0.2215
Psy SM-R, Δ*hopBA1*/Δ*hopAZ1* vs triple hop mutant	0.0731	0.1071

* *p-value* significant at 0.05 level

** *p-value* significant at 0.01 level

REFERENCES

1. Dudnik A, Dudler R: **High-Quality Draft Genome Sequence of *Pseudomonas syringae* pv. *Syringae* Strain SM, Isolated from Wheat.** *Genome Announc* 2013, **1**:e00610–13.

2. Dudnik A, Dudler R: **Non contiguous-finished genome sequence of *Pseudomonas syringae* pathovar *syringae* strain B64 isolated from wheat**. *Stand Genomic Sci* 2013, **8**:420–429.

3. Gardiner DM, Stiller J, Covarelli L, Lindeberg M, Shivas RG, Manners JM: **Genome Sequences of *Pseudomonas* spp. Isolated from Cereal Crops.** *Genome Announc* 2013, **1**:e00209–13.

4. Dudnik A, Dudler R: **Genome and Transcriptome Sequences of *Pseudomonas syringae* pv. *syringae* B301D-R.** *Genome Announc* 2014, **2**:e00306–14.

5. Feil H, Feil WS, Chain P, Larimer F, DiBartolo G, Copeland A, Lykidis A, Trong S, Nolan M, Goltsman E, Thiel J, Malfatti S, Loper JE, Lapidus A, Detter JC, Land M, Richardson PM, Kyrpides NC, Ivanova N, Lindow SE: **Comparison of the complete genome sequences of *Pseudomonas syringae* pv. *syringae* B728a and pv. *tomato* DC3000.** *Proc Natl Acad Sci U S A* 2005, **102**:11064–9.

6. Willis DK, Hrabak EM, Rich JJ, Barta TM, Lindow SE, Panopoulos NJ: **Isolation and Characterization of a *Pseudomonas syringae* pv. *syringae* Mutant Deficient in Lesion Formation on Bean**. *Mol Plant-Microbe Interact* 1990, **3**:149.

7. Bullock WO, Fernandez JM, Short JM: **XL1-Blue: a high efficiency plasmid transforming *recA Escherichia coli* strain with beta-galactosidase selection**. *Biotechniques* 1987, **5**:376–378.

8. Kessler B, de Lorenzo V, Timmis KN: **A general system to integrate *lacZ* fusions into the chromosomes of gram-negative eubacteria: regulation of the Pm promoter of the TOL plasmid studied with all controlling elements in monocopy.** *Mol Gen Genet* 1992, **233**:293–301.

9. Simon R, Priefer U, Pühler A: **A Broad Host Range Mobilization System for In Vivo Genetic Engineering: Transposon Mutagenesis in Gram Negative Bacteria**. *Bio/Technology* 1983, **1**:784–791.

10. Thoma S, Schobert M: **An improved *Escherichia coli* donor strain for diparental mating.** *FEMS Microbiol Lett* 2009, **294**:127–32.

11. Quandt J, Hynes MF: **Versatile suicide vectors which allow direct selection for gene replacement in gram-negative bacteria.** *Gene* 1993, **127**:15–21.

12. Voisard C, Bull CT, Keel C, Laville J, Maurhofer M, Schnider U, D´efago G, Haas D: **Chapter 6. Biocontrol of Root Diseases by *Pseudomonas fluorescens* CHA0: Current Concepts and Experimental Approaches**. In *Mol Ecol Rhizosph Microorg*. Edited by O'Gara F, Dowling DN, Boesten B. Weinheim, Germany: Wiley-VCH Verlag GmbH; 1994:67–89.
